# Seroprevalence of IgA anti Epstein-Barr virus is high among family members of nasopharyngeal cancer patients and individuals presenting with chronic complaints in head and neck area

**DOI:** 10.1371/journal.pone.0180683

**Published:** 2017-08-11

**Authors:** Susanna Hilda Hutajulu, Jajah Fachiroh, Gabriella Argy, Sagung Rai Indrasari, Luh Putu Lusy Indrawati, Dewi Kartikawati Paramita, Theodola Baning Rahayu Jati, Jaap M. Middeldorp

**Affiliations:** 1 Division of Hematology and Medical Oncology, Department of Internal Medicine, Faculty of Medicine, Universitas Gadjah Mada/Dr. Sardjito Hospital, Yogyakarta, Indonesia; 2 Department of Histology and Cell Biology, Faculty of Medicine, Universitas Gadjah Mada, Yogyakarta, Indonesia; 3 Study Program of Medicine, Faculty of Medicine, Universitas Gadjah Mada, Yogyakarta, Indonesia; 4 Department of Otorhinolaryngology Head and Neck Surgery, Faculty of Medicine, Universitas Gadjah Mada/Dr. Sardjito Hospital, Yogyakarta, Indonesia; 5 Field Epidemiology Training Program, Department of Public Health, Faculty of Medicine, Universitas Gadjah Mada, Yogyakarta, Indonesia; 6 Department of Pathology, VU University Medical Center, Amsterdam, The Netherlands; University of North Carolina at Chapel Hill, UNITED STATES

## Abstract

Epstein-Barr (EBV) infection and presence of a nasopharyngeal cancer (NPC) case in the family increases the risk of developing NPC. Aberrant anti-EBV immunoglobulin A (IgA) antibodies (EBV-IgA) may be present in the sera of non-cancer individuals and predict NPC. Limited studies report the presence of EBV-IgA antibodies within non-cancer individuals in Indonesia where the disease is prevalent. This study aimed at exploring whether EBV-IgA was found more frequently among first degree relatives of NPC patients and individuals presenting with chronic symptoms in the head and neck area compared to healthy controls. A total of 967 non-cancer subjects were recruited, including 509 family members of NPC cases, 196 individuals having chronic complaints in the head and neck region, and 262 healthy donors of the local blood bank. Sera were analyzed using a standardized peptide-based EBV-IgA ELISA. Overall, 61.6% of all individuals had anti-EBV IgA reactivity equal to or below cut off value (CoV). Seroreactivity above CoV was significantly higher in females (38.7%) compared to males (28.7%) (p = 0.001). Older individuals had more seroreactivity above CoV (42.5%) than the younger ones (26.4%) (p< 0.001). Seroprevalence was significantly higher in family members of NPC patients (41.7%), compared to 32.7% of individuals with chronic head and neck problems (p = 0.028) and 16.4% healthy blood donors (p< 0.001). As conclusion, this study showed a significant higher seroprevalence in healthy family members of NPC cases and subjects presenting with chronic symptoms in the head and neck area compared to healthy individuals from the general community. This finding indicates that both groups have elevated risk of developing NPC and may serve as targets for a regional NPC screening program.

## Introduction

Nasopharyngeal carcinoma (NPC), a relatively rare cancer in many regions of the world, has a high incidence and mortality in Southeast Asia including Indonesia. Based on local data, its incidence in the country is 6.2 cases per 100,000 population annually, representing the fourth most prevalent cancer in men [1], with most cases being histologically undifferentiated carcinoma [2]. Considering the size of the Indonesian population, about 13,000 new cases of NPC may occur per year, making it a significant national health burden.

The evolution of NPC results from an interaction of genetic factors and environmental influences, including Epstein-Barr virus (EBV) infection. EBV infection has a strong, consistent implication in the pathogenesis of NPC especially the undifferentiated disease [3–5]. The close relationship between EBV and NPC is underscored by the existence of viral DNA, RNA, and protein in all tumor cells, viral reactivation and particular antibodies against EBV antigens in the sera of patients [6,7]. Deviant antibodies against EBV antigens such as viral capsid antigen (VCA), DNase, early antigen (EA), and EBV nuclear antigen 1 (EBNA1) have been proposed for clinical diagnosis [8–11]. We have developed an IgA-EBV diagnostic tool, based on reactivity against EBNA1 and VCA-p18 in one reaction. EBNA-1 is a latent protein expressed in all EBV infected (tumor) cells and responsible for maintenance of the latent EBV episome [12]. VCA-p18 protein is part of the viral capsid, released from virus producing cells [13]. The EBV-IgA ELISA method has been used to select high risk individuals in healthy populations [14] and predicting disease relapse [15]. Multiple studies demonstrated that markedly high EBV-IgA reactivity is detectable in NPC cases preceding clinical symptoms supporting the use of EBV-IgA serology for NPC screening in endemic regions [16–20].

Many reports showed that first-degree relatives of NPC cases are at a higher risk of developing the malignancy when compared to non-family individuals [21–25]. Moreover, family members are considered primary targets for a NPC screening [26–28]. Besides this population, individuals with a history of unexplained chronic nasal, neurological and otological problems have been observed to have risk for NPC development [29–31]. Indeed, patients with NPC frequently present with non-specific symptoms associated with NPC development, such as epistaxis, nasal blockage and discharge, tinnitus and deafness, headaches, diplopia, facial pain and numbness, and swollen lymph nodes in the neck [32].

Frequency of EBV-IgA seroreactivity among healthy individuals has not been extensively analyzed in Indonesia. Our previous work has demonstrated that close family members of NPC cases in the Yogyakarta region had a higher frequency of elevated IgA seroreactivity against EBV-EBNA-1 and -VCA/p18, compared to community controls of NPC cases [14]. Considering that people with chronic problems in the head and neck area may be at risk of developing NPC and may have similar features of immune response against EBV, this study aimed to compare antibody reactivity in the sera of family members of NPC cases with individuals who presented with such complaints. Furthermore, we compare immune response against EBV in both groups versus regional healthy subjects recruited from the local blood bank. Importantly, this approach will determine whether any serological marker differences can be used to identifiy NPC cases among the family and clinical groups compared to healthy controls. This analysis will provide evidence for utility of IgA serology for NPC screening in the general population in Indonesia.

## Methods

### Study population

Data from three studies performed in the Yogyakarta region and coordinated from the Dr. Sardjito Hospital were analyzed for comparison of anti-EBV antibody titers across first-degree relatives of NPC patients, individuals with chronic symptoms in the head and neck areas, and healthy individuals from the local blood bank. The Human Subjects review committee in Yogyakarta approved these projects and informed consent was obtained from all subjects. The family study and the study on patients with chronic head and neck problems were conducted in about the same time period and used similar data collection instruments and same laboratory tests [10,14]. Enrollment of healthy participants from the blood bank was done three years after the two studies finished.

In the family study, 509 first-degree relatives were enrolled and home-visited. Family relatives were defined as fathers, mothers, siblings and off-springs living in the same residence or neighborhood of particular NPC cases. This study was done between year 2008 and 2010. One-hundred and ninety-six subjects with chronic problems in the head and neck area were recruited at the Department of Otorhinolaryngology Head and Neck Surgery, Dr. Sardjito Hospital from July 2006 to December 2010. Recruited subjects came to the department with chronic complaints that lasted for at least 3 months which were not responsive to standard treatment (such as antibiotics, or anti-allergics) for more than 3 times. The clinical symptoms included rhinitis, nasal obstruction, epistaxis, tinnitus, hearing impairment, ear discomfort (sensation of fullness or pain), headache, facial pain, dizziness and dysphagia. These patients were selected for having symptoms in the spectrum characteristics of NPC in early disease [2]. Two-hundred and sixty-two healthy individuals were enrolled from the blood banks of City of Yogyakarta and Sleman District, Yogyakarta, in 2013. These healthy subjects had resided in the province at least for five years and had no recorded symptoms of any malignancy nor chronic disease in the head and neck region and had no family history of NPC. Demography data including age and sex were also included.

### Ethics approval and consent to participate

The Human Subjects review committee in Faculty of Medicine, Universitas Gadjah Mada, Yogyakarta, Indonesia (committee’s reference numbers: KE/FK/58/EC and KE/FK/336/EC) approved these projects and informed consent was obtained from all subjects prior to any data acquisition, sample collection, or data analysis.

### Blood sample and EBV serology

Blood sampling was done from the arm vein in all subjects. For individuals who rejected having venous blood drawn, blood was taken using a finger prick method as described previously [33]. Serology analysis was performed by ELISA using a combination of two EBV antigens, i.e. EBNA1 as viral latent antigen and VCA-p18 as lytic antigen. Immunoglobulin A (IgA) reactivity against EBNA1 plus VCA-p18 was analyzed in ELISA using a peptide-based antigen combination.(10) A positive result was defined as optical density (OD)_450_> 0.354, as defined before and all procedures and definition of CoV were characterized elsewhere [10]. This assay showed a good sensitivity (90.1%) and specificity (85.4%) in discriminating NPC cases from non-cancer controls. Further the assay is referred as EBV-IgA ELISA.

### Statistical analysis

The difference of distribution of EBV antibody status among three different studies was analyzed using the Pearson’s χ^2^ test. To evaluate possible demographic determinants of EBV-IgA seroreactivity, factors including age and gender were also compared using the Pearson’s χ^2^ test. Continuous variables were tested using the Mann-Whitney U-test. All tests were obtained using SPSS 24. All p-values presented were two-sided with p≤ 0.05 defined as statistically significant.

## Results

A total of 967 non-cancer subjects were recruited. The ages of all participants ranged from 18–80 years. The characteristics of subjects among different populations are shown in Table 1. The family study group and patients with chronic head and neck complaints were dominated by females (51.7% and 60.7%, respectively), while the group of healthy participants from the blood bank was dominated by males (87.0%) (p< 0.001). Older individuals aged >40 years were prominent in the family study group (52.3%), while younger participants dominated the other two groups (60.2% and 80.5%, respectively) (p< 0.001). Overall, OD_450_ values of anti-EBV IgA (seroreactivity) of all subjects ranged from zero to 3.408. There were 648 out of 967 (61.7%) individuals having anti-EBV IgA antibody titer equal or below CoV and 319 (30.4%) individuals showing reactivity above CoV. Frequecy of seroreactivity above CoV (seroprevalence) was significantly higher in females (161/416; 38.7%) compared to males (158/551; 28.7%) (p = 0.001), with mean ± standard of deviation (SD) of 0.407±0.592 and 0.521±0.683, respectively. When discriminated by age using 40 years as a CoV [2,34], the older group showed significantly higher seroprevalence (168/395; 42.5%) than the younger group (151/572; 26.4%) (p< 0.001), with mean ± SD of 0.591±0.773 and 0.363±0.499, respectively.

**Table 1:** Characteristics of subjects across different groups (n = 967).

	First-degree relatives of NPC cases (n = 509) n (%)	Subjects with chronic problems in head and neck (n = 196) n (%)	Subjects from the local blood bank (n = 262) n (%)	P value
Sex				
Males	246 (48.3)	77 (39.3)	228 (87.0)	< 0.001
Females	263 (51.7)	119 (60.7)	34 (13.0)	
Age				
≤40 years	243 (47.7)	118 (60.2)	211 (80.5)	< 0.001
>40 years	266 (52.3)	78 (39.8)	51 (19.5)	
EBV-IgA values				
Mean level ± SD	0.550±0.722	0.440±0.616	0.284±0.383	
Median value	0.289	0.232	0.152	
Range of value	0.001–3.408	0.000–3.380	0.001–0.249	
EBV-IgA*				
≤CoV (seronegative)	297 (58.3)	132 (67.3)	219 (83.6)	< 0.001
>CoV (seropositive)	212 (41.7)	64 (32.7)	43 (16.4)	

*Normalized values

**Fig 1:**
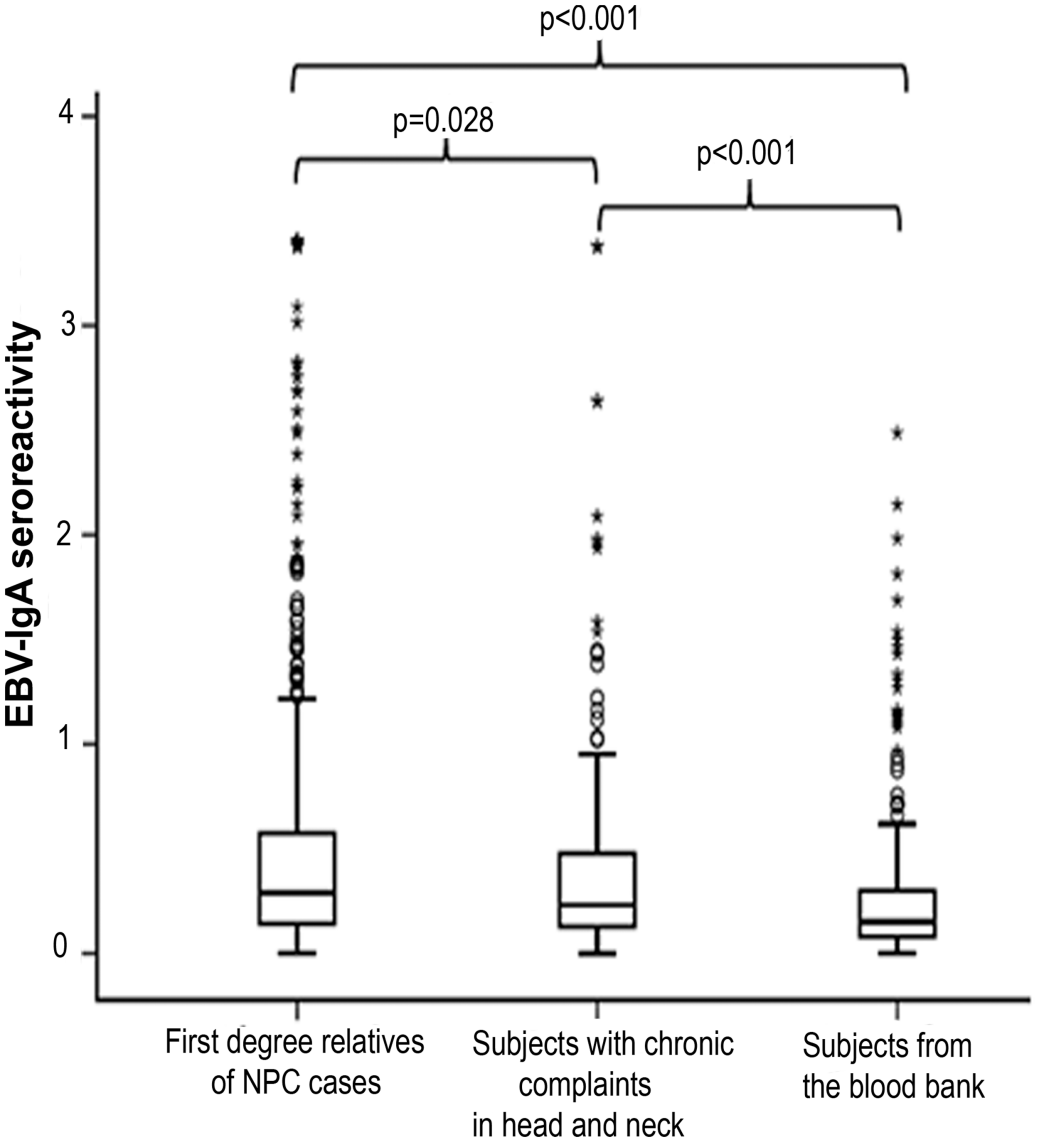
Boxplot of normalised EBV-IgA ELISA seroreactivity by groups. Seroprevalence was shown by 41.7% of family members of NPC cases, compared to 32.7% of individuals with chronic problems in the head and neck area and 16.4% of individuals recruited from the blood bank (Pearson’s χ^2^ test p< 0.001). A significant difference in seroprevalence was also found between NPC family members and patients with chronic diseases (p = 0.028). Extreme and mild outliers were represented by symbols of * and °.

The median values of seroreactivity against EBV in the sera of the three populations were 0.289, 0.232, and 0.152, respectively. There were few differences between median values of seroreactivity in the NPC family member group and in subjects with chronic head and neck complaints (p = 0.062). Significant differences were observed when comparing median values of seroreactivity in the groups of NPC family members and patients with chronic disease with blood bank donors (p values for both comparisons <0.001) (Table 1). Fig 1 demonstrates a boxplot of normalised EBV-IgA ELISA seroreactivity in the three groups.

EBV-IgA seropositivity was shown by 212 family members of NPC patients (41.7%), compared to 64 individuals with chronic head and neck problems (32.7%) and 43 healthy individuals (16.4%) recruited from the blood bank (p<0.001). A significant difference in seroprevalence was also found between NPC relatives and patients with chronic head and neck problems (p = 0.028).

Table 2 shows the distribution of EBV-IgA seroreactivity by sex and age in each group. In all groups seroprevalence was higher in females and older subjects than their counterparts. The difference in seroprevalence between genders in the three groups was small. For age classification, significant differences were observed in subjects presenting with chronic complaints (p = 0.008) and community healthy subjects (p = 0.005).

**Table 2:** Distribution of seroprevalence by sex and age in the three groups.

Groups	Number	Number (%) positive	P value
**First-degree relatives of NPC cases**	509		
Sex			
Males	246	101 (41.9)	0.793
Females	263	111 (42.2)	
Age			
≤40 years	243	93 (38.3)	0.139
>40 years	266	119 (44.7)	
**Subjects with chronic problems in head and neck area**	196		
Sex			
Males	77	21 (27.3)	0.196
Females	119	43 (36.1)	
Age			
≤40 years	118	30 (25.4)	0.008
>40 years	78	34 (43.6)	
**Subjects from the local blood bank**	262		
Sex			
Males	228	36 (15.8)	0.481
Females	34	7 (20.6)	
Age			
≤40 years	211	28 (13.3)	0.005
>40 years	51	15 (29.4)	

To analyze the elevated antibody levels observed among these non-cancer populations, the distribution of seropositivity by gender and age across the three populations was further evaluated (Table 3). Data showed that for both sexes and age classifications, seroprevalence was higher in the group of family members of NPC cases and subjects who presented with chronic head and neck symptoms compared to healthy participants in general community. Significant differences were shown for all determinants except older age.

**Table 3:** Distribution of seroprevalence by sex and age across three groups.

	First-degree relatives of NPC cases (n = 509) n (%)	Subjects with chronic problems in head and neck area (n = 196) n (%)	Subjects from the local blood bank (n = 262) n (%)	P value
Male sex	101/246 (41.1)	21/77 (27.3)	36/228 (15.8)	< 0.001
Female sex	111/263 (42.2)	43/119 (36.1)	7/34 (20.6)	0.041
Age of ≤40 years	93/243 (38.3)	30/118 (25.4)	28/211 (13.3)	< 0.001
Age of >40 years	119/266 (44.7)	34/78 (43.6)	15/51 (29.4)	0.125

## Discussion

Nasopharyngeal carcinoma (NPC) is characterized by a late clinical presentation due to the hidden site of primary tumor formation and non-specific symptoms at an early stage. Disease diagnosis at the preclinical stage is of crucial importance to improve treatment efficacy and patient survival. The consistent presence of EBV components especially among NPC in endemic populations has pushed for the development of EBV-based diagnostic methods. Regardless of the development of technology in detecting EBV DNA from tissue or peripheral blood of NPC patients, the use of non-invasive serology is more suitable for screening purpose due to its high predictive value and its cost efficiency [35,36]. Regular serological follow-up to observe dynamic fluctuations of anti EBV-IgA in time is recommended for a NPC secondary prevention program [19,37,38]. The current study provides the first data on IgA seroreactivity against EBV antigens in non-cancer subjects from Indonesia, where NPC is a prevalent malignancy [2]. However, the multiple ethnicities in Indonesia may interfere with a homogenicity pattern of seropositivity. Results from this study provide a first basis for NPC screening in Indonesia.

Zou et al. reported that an individual with family history of NPC had 14.2 times higher risk of NPC development compared to individuals without a family history of NPC [30]. Pickard et al. employed IgA-VCA, DNase neutralizing activity, and IgA-EBNA1, and showed that seroprevalence in unaffected family members of NPC cases was 5–6 times significantly higher than in members of the community. The report showed that IgA-VCA, DNase neutralizing antibody, and IgA-EBNA-1 among unaffected family members of NPC were 28.4%, 11.8% and 20.1%, respectively [39]. However, for positivity to any of these three markers, the seroprevalence increased to 47.9%. This proportion was similar to our previous finding (41.7%) in local populations using combined VCA-p18/EBNA-1 IgA-detection in one ELISA test [14]. This result further confirms that the use of multiple antigens of EBV provided better detection [10,40].

In this current study we observed that EBV-IgA seroprevalence in family members of NPC was significantly higher than that of normal healthy subjects without a family link to NPC (41.7% versus 16.4%, p<0.001). This finding deviates from our previous results showing similar frequency of seroreactivity between first degree relatives of NPC cases and healthy matched controls living in the neighborhood (41.2 versus 39.2%, p = 0.770) [14]. More healthy participants (n = 967) recruited in the present study compared to our previous study (n = 86) may affect this difference. Healthy population-based controls living in the same area with NPC cases [14] might share environmental exposures with the NPC cases, leading to rather high seroreactivity in the population previously reported. Moreover, different recruitment protocol in the present study might contribute to this discordance since all healthy subjects enrolled were more strictly selected by infection screening and clinical eligibility.

Late clinical presentation of NPC is influenced by unspecific disease symptoms. Multiple head and neck complaints are observed in the early clinical stages of NPC [32]. Ekburanawat et al. (2010) and Zhou et al. (2000) showed that past history of chronic nasal and/or otological disease were related to NPC risk by showing an odds ratio (OR) of 2.71 (95% confidence interval/CI = 1.45–5.06) and 3.6 (95% CI = 1.3–10.1), respectively [29,30]. These studies additionally demonstrated that the symptoms might be simultaneously observed with elevated titer to anti-EBV IgA [19]. The inflammatory and other particular benign conditions in such areas can further predispose the nasopharyngeal mucosa to transformation on exposure to carcinogenic substances in the environment [29]. Our findings showed that seroprevalence in subjects with chronic complaints in head and neck area was significantly higher than that of healthy subjects, indicating that these individuals are also suitable targets for early detection program in our region. Moreover, seroprevalence in this group was significantly lower than that of first degree relatives of NPC patients. This result can be affected by close genetic association and similar environmental exposure among family members.

In a NPC endemic region, most healthy populations are considered to have a latent state of EBV infection, marked by low or absent anti-EBV IgG or IgA titer, respectively. Upon exposure to EBV-lytic activation substances (nitrosamine, UV, butyrates, phorbol esters), local immune suppression, or glucocorticoid-inducing stress, EBV might be driven to enter the lytic state [41–45]. This process might be reflected by elevated IgG or IgA anti EBV titers [19].

Within the total of our study populations, we observed that seroprevalence was higher among females and those of older age, which is in agreement with Du et al. (2016) [38]. It is assumed that older subjects had more exposure to EBV triggers along with weakening of immune response which may facilitate EBV reactivation, leading to elevated IgA anti EBV. Some reports demonstrated that seroreactivity against EBV in females was stronger than in males, possibly due to hormonal effects on immunologic responses [46,47].

Cohort studies for early detection of NPC have been reported recently involving populations in Southern China and Taiwan [19,35,37,48]. In Indonesia issues are still being raised regarding populations to target and suitable screening methods. This study confirms that in Yogyakarta region seroprevalence of IgA anti EBV is higher among NPC relatives and individuals presenting with chronic symptoms in the head and neck area compared to regional healthy individuals. It is also proposed that the combined analysis of IgA to [VCA-p18+EBNA-1] in one assay may be suitable for NPC screening in Indonesia.

## Conclusion

This study demonstrated a significant higher seroprevalence in healthy family members of NPC cases and subjects presenting with chronic complaints in the head and neck area compared to healthy subjects. Defined as high responders, these groups of individuals are considered as candidates for an NPC screening program.
